# Evaluating Autophagy in Preimplantation Embryos

**DOI:** 10.1080/27694127.2022.2101335

**Published:** 2022-07-21

**Authors:** Zuleika C. L. Leung, Hailey L. M. Hunter, Basim Abu Rafea, Andrew J. Watson, Dean H. Betts

**Affiliations:** a Department of Obstetrics and Gynaecology; b Department of Physiology and Pharmacology, The University of Western Ontario, London Ontario, Canada, N6A 5C1; c The Children’s Health Research Institute – Lawson Health Research Institute, London, Ontario, Canada, N6C 2R5

**Keywords:** Autophagy, ATG, embryonic stem cells, LC3, preimplantation embryo

## Abstract

Autophagy is an essential cellular degradation pathway in the mammalian preimplantation embryo. In this review, the process of autophagy is outlined and its function pertaining to preimplantation embryos is discussed. The current methods of measuring autophagy in preimplantation embryos are also summarized, with highlights of advances and challenges that one may encounter when examining preimplantation embryo autophagy. This article identifies the techniques, assays, and available models that are viable but infrequently used in the context of examining autophagy in preimplantation embryos. We aim to bring attention to alternative assays that could contribute to future analysis of autophagic pathways in preimplantation embryos.

**Abbreviations:** AMBRA1: Activating molecule in Beclin-1-regulated autophagy 1; ATG: Autophagy-related protein; AL: Autolysosome; BCL-2: B-cell lymphoma 2; BECN1: Beclin-1; CQ: Chloroquine; ER: endoplasmic reticulum; ES cells: Embryonic stem cells; GFP: Green fluorescent protein; ICM: Inner cell mass; IF: Immunofluorescence; LC3: Light chain 3; LC3-II: Light chain 3 (lipid-conjugated); MAPK: mitogen-activating protein kinase; mTOR: Mammalian target of rapamycin; p62/SQSTM1: Sequestome 1; PA: Palmitic acid; PG: Phagophore; PtdIns3K: Phosphatidylinositol 3-kinase; PtdIns3P: phosphatidylinositol-3 phosphate; qPCR: Quantitative polymerase chain reaction; RFP: Red fluorescent protein; ROS: Reactive oxygen species; SNARE: Soluble N-ethylmaleimide-sensitive factor attachment protein receptor; TS cells: Trophoblast stem cells; ULK1: UNC-51-like kinase 1; WB: Western blot

## 1. Introduction

Autophagy is a cellular degradation pathway that is highly conserved in eukaryotes [1]. In brief, it is a pathway in which cytoplasmic components in the cells are degraded by lysosomes for reuse in other cellular functions [1]. Autophagy is widely utilized by mammalian organisms, from preventing heteroplasmy by degrading paternal mitochondrial DNA [2] to the survival of fetuses during early starvation period and pregnancy [3,4]. At a cellular level, macroautophagy (thereinafter referred to as autophagy) serves many different functions, including organelle clearance, regulation of development, as well as stress adaptation [5]. Previous studies have identified autophagy as an essential component in regulating cellular homeostasis [6], cell growth, and preimplantation embryo development [7]. Autophagy also serves as a protective mechanism against cytotoxic environments in various cell types, including early embryos [8–13]. Although autophagy is generally beneficial for overall cell homeostasis, it must be balanced at optimal levels as disruption of autophagic processes contributes to cell death [14]. In this review, the process of autophagy is extensively described and its function pertaining to preimplantation embryo development is summarized. The current methods of measuring autophagy in preimplantation embryos are also discussed.

## 2. Autophagy

**Figure 1.**
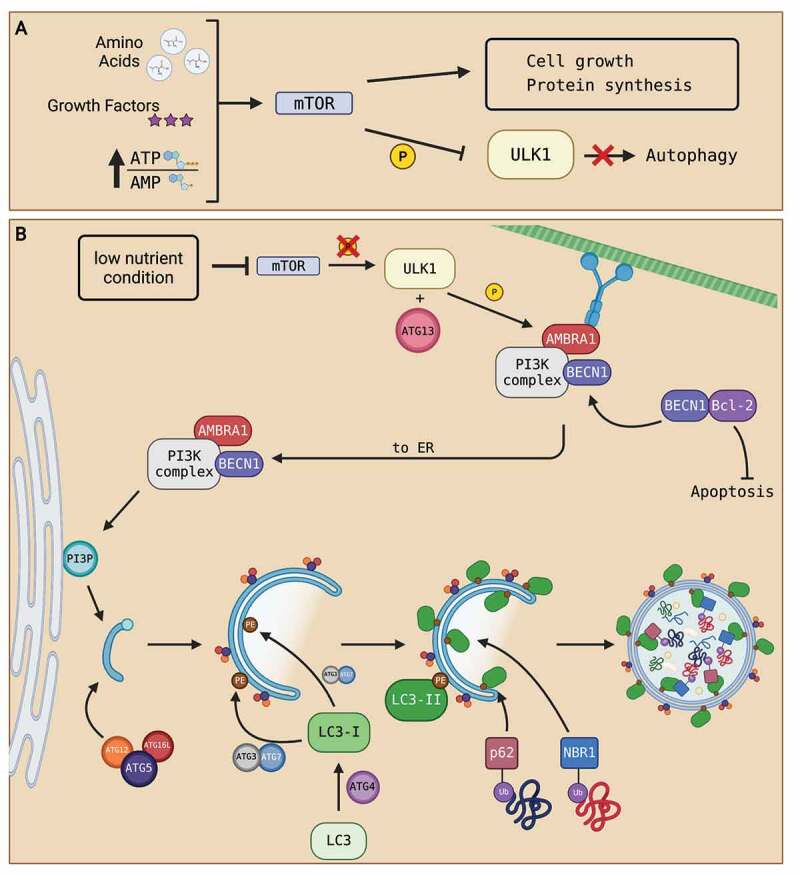
Summary of autophagosome formation. (A) Under nutrient-rich conditions (high abundance of amino acids, growth factors, and a high ATP:AMP ratio), cell growth signals converge to activate mTOR. The activation of mTOR phosphorylates ULK1 complex and deactivates it, resulting in the inhibition of autophagy. (B) Under nutrient-deprived conditions, mTOR activity is suppressed. Inhibition of mTOR prevents phosphorylation of ULK1 complex which activates autophagy. Activated ULK1, along with ATG13, phosphorylate downstream PtdIns3K complex subunits, like BECN1 and AMBRA1. The activation of autophagy dissociates BECN1 from BCL-2, freeing BCL-2 to prevent apoptosis and allow BECN1 to join AMBRA1 and the rest of the PtdIns3K complex located at the microtubule network. The phosphorylation of AMBRA1 frees the entire PtdIns3K complex from dynein motor unit at the microtubule network to allow translocation to the ER. Once at the ER, the PtdIns3K complex produces PtdIns3P that initiates the nucleation of a PG. Then, the ATG5-ATG12-ATG16L1 complex associates and attaches to the PG as the PG elongates to form an autophagosome. The ATG5-ATG12-ATG16L1 complex then recruits the binding of LC3-II to both the outer and inner membranes of the autophagosome. Processing of LC3 via ATG proteins and lipidation with PE on the autophagosome membranes transform LC3 into LC3-I then LC3-II upon lipidation. LC3-II proteins on the inner membrane recruit proteins like p62 and NBR1 that are attached to ubiquitinated proteins to bring in autophagosome contents. As contents are directed into the PG, elongation of the membrane eventually seals the membrane and creates an autophagosome. Created with BioRender.com.

Autophagy is an evolutionarily conserved degradation pathway in eukaryotic cells [1]. It is widely recognized as a pro-survival mechanism due to its protective functions and its involvement with mediating cell stress, like nutrient starvation and reactive oxygen species (ROS) buildup [8,15]. Under normal conditions, autophagy induction is negatively regulated by the protein kinase mTOR (mammalian target of rapamycin), in which mTOR phosphorylates its downstream ULK1 (UNC-51-like kinase 1) complex to block autophagy initiation [8]. Nutrient-rich environments (high abundance of amino acid and energy constituents) and growth factor signaling converge on mTOR to promote cellular growth [16]. A summary of the process of autophagy inactivation is displayed in Figure 1A. The common consensus in the literature supports that autophagy is generally mTOR-dependent. However, other mechanisms of activating autophagy, independent of mTOR, are also proposed in various cell types [17–19]. Below, we outline the three-step process of autophagy consisting of initiation, maturation, and degradation of an autophagosome to recycle cellular components for other cellular functions.

### 2.1. Initiation of autophagy: autophagosome formation

Under nutrient-deprived or stress conditions, mTOR activity is suppressed [8]. For example, low energy condition, as reflected by a high AMP-to-ATP ratio, results in the inhibition of mTOR by AMPK (AMP kinase), halting cell growth and activating autophagy [8]. Inhibition of mTOR dissociates and dephosphorylates the downstream ULK1 complex while direct phosphorylation of ULK1 by AMPK activates ULK1 complex [20]. Activated ULK1 complex consisting of ATG- (autophagy-related protein) 13 and other proteins, phosphorylate and activate downstream class III phosphatidylinositol 3-kinase (PtdIns3K) protein complex [21]. The PtdIns3K complex consists of factors like BECN1 (Beclin-1) and AMBRA1 (activating molecule in beclin-1-regulated autophagy 1). AMBRA1 is the connector protein that binds BECN1 and PtdIns3K at the dynein complex until autophagy is initiated [21]. The activation of ULK1 phosphorylates AMBRA1 to allow translocation of the PtdIns3K complex to the endoplasmic reticulum (ER) for autophagosome formation [22]. Additionally, AMBRA1 regulates autophagy by stabilizing the activity of ULK1 [23]. BECN1 is an important mediator for autophagy as the chemically-induced cleavage of BECN1 inhibits autophagy [24]. The activation of BECN1 dissociates itself from BCL-2 (B-cell lymphoma 2), resulting in the activation of BCL-2 for anti-apoptotic functions to support the pro-survival nature of autophagy [25]. The activation of the PtdIns3K complex produces PtdIns3P (phosphatidylinositol-3 phosphate), which initiates the nucleation of a phagophore (PG), creating a phagophore assembly site at the ER [26]. The elongation of the PG involves the recruitment of lipids to extend and form a double membrane autophagosome. Melia and colleagues [21] estimated that over 100 million lipid molecules are required per cell to support autophagosome formation. The presence of PtdIns3P on the autophagosome membrane also recruits protein complexes for membrane elongation [21]. One such example is the ATG5-ATG12-ATG16L1 (ATG5-12-16) complex. The ATG5-12-16 complex is recruited to specific sites on the autophagosome membrane and these complexes contribute to autophagosome curvature and recruitment of lipid-conjugated microtubule-associated protein light chain 3 (LC3-II) [21,27]. ATG5-12-16 complex contributes to ATG3 activity in lipid conjugation of light chain 3 (LC3) [28]. LC3 is first translated in the cell as a precursor. Through ATG4 processing at the carboxylic terminal, LC3 transforms into its cytosolic form LC3-I [29]. With ATG3 and ATG7, a PE (phosphatidylethanolamine) on the autophagosome membrane is conjugated onto LC3-I to create the lipid-conjugated form, LC3-II, which is then become attached onto autophagosome membranes upon lipidation [28,29]. LC3-II is a marker for autophagosome formation as it binds to both the inner and outer membranes of the autophagosome [30]. LC3-II is widely accepted as a marker for autophagosome formation because its generation is initiated by autophagy induction and the protein is degraded along with autophagosome contents [31]. Elongation of the PG continues until the closure of the membranes. Although evidence is limited, the closure of the membranes has been shown to occur through the endosomal sorting complex required for transport (ESCRT) machinery [32,33]. PG membrane-bound Rab5 GTPases recruitment of ESCRT-III enables subunits like vacuolar protein sorting 4 (Vps4) ATPase to catalyze membrane fission, creating an autophagosome [32,33].

Autophagosome contents are quite random and non-discriminatory [1]. However, some selective degradation processes have been identified; for example, through p62/SQSTM1 (sequestome 1) adaptor proteins and NBR1 (neighbour of BRCA1 gene 1). LC3-II in the inner membrane can interact with and recognize p62/SQSTM1 that bring in ubiquitinated proteins for degradation [34]. Additionally, NBR1 directs ubiquitinated protein aggregates into autophagosomes by binding to LC3-II of the inner membrane, independent of the p62 mechanism [35]. A summary of the autophagosome formation process is shown in Figure 1B.

### 2.2. Autophagosome maturation: autolysosome

After the completion of the autophagosome, the autophagosome must fuse with a lysosome for content degradation. To do so, autophagosomes must dissociate from the ER to the cytoplasm to interact with a lysosome. The transport of an autophagosome in a mammalian cell requires the cytoskeletal microtubules, which with the help of FYVE and coiled-coil domain containing 1 (FYCO1) and dynein units, move autophagosomes towards the lysosomes [36–39]. The disruption of microtubules has been previously found to lower autophagosome maturation [40], suggesting an indispensable role for microtubule networks in autophagosome transport. Two different hypotheses are proposed for the attachment of autophagosomes to microtubules. One being that LC3-II on the outer autophagosome membrane directly binds onto microtubules, as antibodies against LC3-II N-terminus prevented autophagosome trafficking towards lysosomes [38]. Another hypothesis is that Rab7, a GTPase protein located on the late endosomes and lysosomes, recruits the dynein motor units attached to autophagosomes and thus contributes to its transport towards lysosomal structures [41]; however, evidence is scarce.

**Figure 2.**
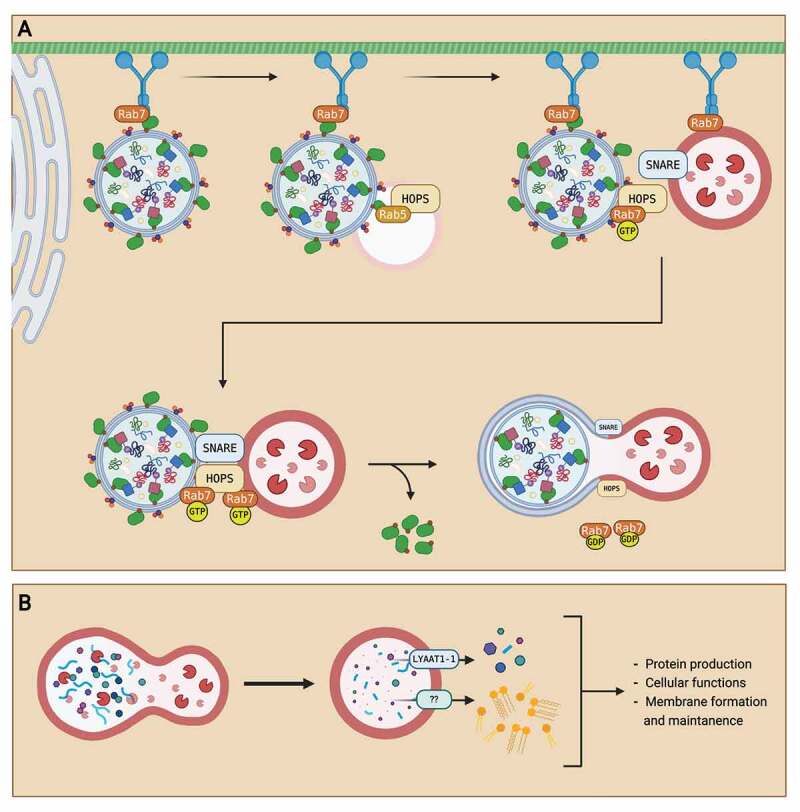
Summary of autophagosome maturation and degradation. (A) After autophagosome formation, the autophagosome is transported from the ER to the lysosome for maturation into an autolysosome. An autophagosome is transported through the cell via the microtubule network with a dynein motor protein. The autophagosome is first fused with an early endosome. Then, the autophagosome is transported towards the lysosome as Rab7 GTPase on the lysosome recruits the dynein protein. When the autophagosome is in near proximity to the lysosome, Rab7 GTPase localizes to the HOPS complex and recruits SNARE proteins to attach the autophagosome with the lysosome. After attachment, hydrolysis of the GTP to GDP on the Rab7 GTPase provides energy for the fusion of autophagosome and lysosome, creating an autolysosome. (B) After the fusion event, autophagosome contents are degraded into monomeric units by lysosomal enzymes. The monomeric units are then transported out of the autolysosome via transporters like LYAAT-1. The monomeric units released into the cytosol are then reused for other cellular functions, like protein production, membrane formation, and cell maintenance. Created with BioRender.com.

The autolysosome (AL) defines the structure of the autophagosome after lysosome fusion. This fusion occurs in a stepwise fashion, with the acquisition of different lysosomal membrane proteins and hydrolases at each step [36]. First, autophagosomes fuse with early endosomes. Fusion of autophagosomes with early endosomes is required prior to fusion with lysosomes for proper content degradation to occur during autophagy [42]. This fusion, however, does not result in the formation of an acidic lumen nor the necessary proximity for degradation [42]. Next, autophagosomes fuse with late endosomes and lysosomes. The fusion between autophagosomes and lysosomes requires Rab GTPases, membrane-tethering proteins, and SNARE (soluble N-ethylmaleimide-sensitive factor-attachment protein receptor) proteins [36]. Rab7 protein is the main coordinator of the fusion process in which it localizes onto specific sites of the autophagosome membrane [36]. Then, membrane-tethering proteins called HOPS (homotypic fusion and vacuolar protein sorting) complex are recruited to Rab7 sites to attach the SNARE proteins on both the autophagosome and lysosome for the fusion event [43]. The interaction of the SNARE proteins brings the autophagosome and lysosome together, allowing the fusion event to create the AL. A summary of the process of autophagosome maturation is presented in Figure 2A. Autophagosome degradation

### 2.3. Autophagosome degradation

After fusion of the autophagosome with a lysosome, autophagosome contents are degraded by lysosomal hydrolases. This degradation process degrades organelles and proteins into monomeric units like lipids and amino acids [15]. The degraded contents are then exported out of the lysosomes, into the cytoplasm, for reuse in other cellular functions [15]. Little is known about lysosomal transporters in mammalian cells. So far, only the LYAAT-1 (lysosomal amino acid transporter 1) has been identified for the exportation of amino acids from the lysosome to the cytoplasm [44]. A summary of the process of autophagosome degradation is displayed in Figure 2B.

## 3. Autophagy in preimplantation embryos

### 3.1. The role of autophagy in preimplantation embryo development

Preimplantation embryo development defines the period of growth from a zygote to the attachment of a blastocyst to the uterine wall [45]. Autophagy was first reported to be essential for preimplantation development of mammalian embryos by Tsukamoto and colleagues [7]. The authors reported that oocyte-specific knockout of *Atg5* in mouse embryos results in developmental arrest at the 4- to 8-cell stage *in vitro* [7]. Furthermore, autophagy-deficient mice (*atg5*^−/−^ mice) survive through preimplantation and prenatal periods but do not survive past one day of delivery, possibly due to maternally inherited ATG5 proteins [7]. Tsukamoto et al. [7] also identified that autophagy is induced by fertilization and then briefly suppressed until the 2-cell stage. This notion was further extended by Yamamoto et al. [19] as they attributed this fertilization-induced autophagy to be independent of mTOR. It was proposed that such autophagy activation results in an indirect activation of ULK1 and it only lasts approximately 20 hours post-fertilization in mouse embryos [19]. Regardless, fertilization-induced autophagy is important for zygotic genome activation (ZGA) where maternal proteins are degraded as zygotic proteins are synthesized [7]. Our group recently characterized the relative mRNA transcript level of autophagic markers in mouse preimplantation embryos at different developmental stages. The expression of *Lc3b* increased gradually as development progressed from the 1-cell stage towards the blastocyst stage [46], suggesting that embryos were slowly primed for autophagy throughout development to enable autophagy activation at the blastocyst stage. Consistently, *Becn1* levels were significantly increased at the blastocyst stage [46]; trophoblast and inner cell mass (ICM) of late-stage blastocysts also express an elevation in various autophagy markers [47]. Immunofluorescent LC3 puncta counts per cell reflecting the number of autophagosomes at each embryonic stage also supports this notion [46]. Overall, it is evident that autophagy is induced after fertilization, briefly suppressed until the 2-cell stage, then gradually increase throughout preimplantation development until the late blastocyst stage. Furthermore, an optimal balance in autophagy is critical in early preimplantation embryo development. Lee et al. [48] applied autophagy modulators to investigate their effects on preimplantation development in mouse embryos. Both autophagy inducers and autophagy inhibitors ultimately disrupt normal levels of autophagy, leading to apoptosis and disruption of blastocyst development [48]. The imbalance in autophagic mechanisms can affect developmental competency [7], cell fate determination [49], and even survival through cell stress or nutrient-deprived environments [1].

### 3.2. Autophagy in stress adaptation

During embryogenesis, the growing embryo experiences various types of stress. The source of local stress on the embryo during normal development includes oxidative stress and inflammation among many others. Whereas environmental stress factors include aberrant exposure to maternal hormones and toxins [50] from conditions like maternal diabetes. Embryo manipulation and growth *in vitro* also pose additional stress during embryogenesis through handling and culture conditions.

One of the main factors for stress during embryogenesis is energy expenditure. A large amount of energy is required to support the exponential growth and division during the early cleavage stages. After fertilization, the developing embryo at the early stages utilizes energy from mitochondrial oxidative phosphorylation of pyruvate [51]. Pyruvate is supplied by surrounding cumulus cells and follicular fluid to be taken up into the mitochondria [52]. It is not until after compaction at the 8-cell stage, in the mouse, that energy production from glucose via glycolysis is favored over pyruvate oxidation, with an accompanying increase in oxygen consumption [53]. Preimplantation embryo oxygen consumption is significantly lower in the early cleavage stages compared to the blastocyst stage, indicative of a low level of aerobic metabolism at the early stages [54]. The quiet embryo hypothesis proposed by Leese [55] suggests that instead of inactivity, mitochondria of the embryo at early stages function at a minimal level to provide just enough energy to meet its energy requirements. This hypothesis has been widely accepted as it nicely provides a sound reason behind low energy metabolism in early embryos – to minimize ROS production from direct pyruvate oxidation [52]. ROS is an oxygen substrate that can cause oxidative damage to organelles and DNA. A study by Dumollard et al. [51] reported that a high pyruvate environment in culture is negatively correlated to blastocyst development of mouse embryos. It is proposed that the excess source of the energy metabolite, pyruvate, may contribute to ROS production that disrupts blastocyst development [51].

Although embryos can utilize lipids as a source of energy metabolites [52], a high-fat environment is detrimental to preimplantation embryo development. Follicular fluids of mothers with high BMI levels tend to have higher concentrations of free fatty acids and triglycerides [56]. High lipid contents taken up by the oocytes from the follicular fluid produce ROS and lipid peroxides that lead to lipotoxicity [57]. Many organelles including the mitochondria and ER experience structural remodeling and dysfunction due to lipotoxicity, which can ultimately induce apoptotic cell death [58]. Our previous report by Yousif et al. [59] showed that elevated levels of palmitic acid (PA) treatment to mouse preimplantation embryos increase gene expression of ER stress pathway constituents at the PERK and IRE1α arm. Our group also presented that mouse preimplantation embryo autophagy is impacted after exposure to elevated levels of non-esterified fatty acids. Specifically, exposure of mouse embryos to elevated levels of PA heighten autophagosome formation and lower lysosomal activity, which ultimately impairs autophagosome degradation [46]. It is therefore evident that high lipid contents induce cellular stress in preimplantation embryos which complicates autophagic mechanisms.

## 4. Current Methods of measuring autophagy in preimplantation embryos

Studies have extensively employed various techniques and assays to measure the level of autophagy in preimplantation embryos. Direct measurement of autophagic levels reveals important status of the embryo during development and even during cell stress events. However, this direct measurement of autophagy could only provide a “snapshot” of the autophagic level at an instance. The elevated presence of an autophagic marker could indicate an increase of autophagosome formation or a decrease in autophagosome maturation and degradation, or both. On the contrary, a reduction of an autophagic marker could indicate a lowered level of autophagosome formation or an increase in autophagosome maturation and degradation, or both. It is thus, important that not only should autophagy be directly measured but it should also be evaluated at each step of the autophagic pathway. 3-methyladanine (3-MA), bafilomycin A1, and chloroquine (CQ) are common inhibitors employed in the literature, among many others for this purpose [60]. 3-MA halts autophagy at the early stages by inhibiting PtdIns3K activity [61]. Although 3-MA is an effective autophagy inhibitor, the employment of 3-MA has also shown to produce off-target effects, inhibiting other kinases like the mitogen-activating protein kinases (MAPK) that are involved in other cellular processes [60,62]. Other inhibitors of autophagy at the early stages include inhibitors of vacuolar protein sorting 34 (Vps34) enzyme of the PtdIns3K complex like VPS34-IN1 and ULK1 inhibitors like MRT68921 [63,64]. On the other hand, inhibitors that prevent autolysosome maturation and degradation can also be employed in evaluating changes in autophagy levels. Bafilomycin A1 inhibits lysosomal proton transport, hence disrupting the acidic environment of the lysosome and preventing the degradation of autophagosomes [60,65]. Similarly, CQ neutralizes lysosomal pH and disrupt lysosomal function, thereby inhibiting autophagosome degradation [60]. It was previously found that CQ disrupts the endo-lysosomal pathway that impairs autophagosome-lysosome fusion; however, this disruption accompanies the disorganization of the golgi complex that ultimately produces cytotoxic effects [66]. Furthermore, CQ has been previously reported to induce LC3 lipidation in an non-canonical manner, one that is similar to LC3-associated phagocytosis (LAP) which involves single-membraned vesicles [67,68]. Despite these limitations of CQ usage in *in vitro* evaluation of autophagy, it is currently the only FDA-approved drug among the other inhibitors and hence it is commonly used in the literature.

**Figure 3.**
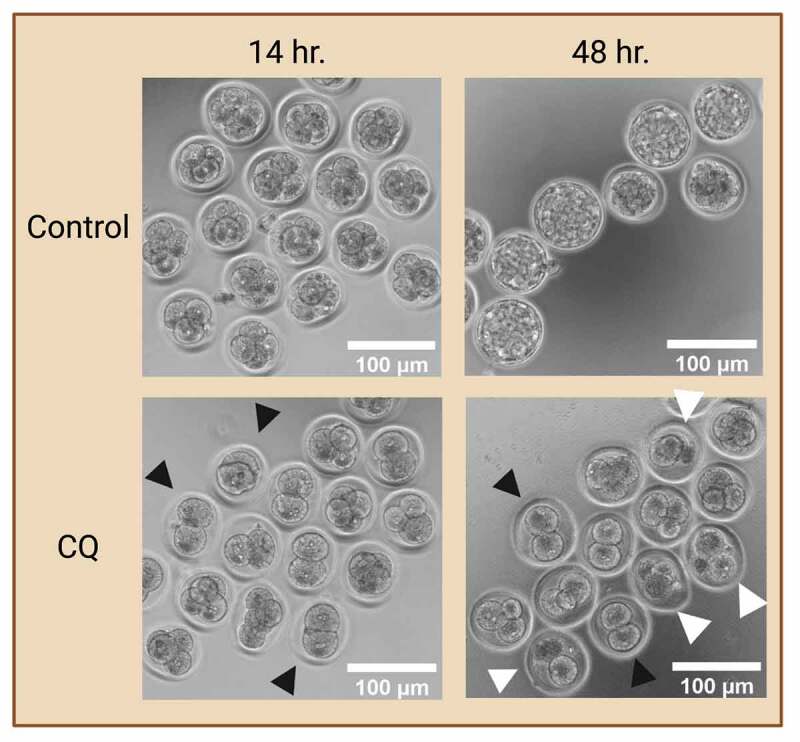
Preimplantation embryo morphology after exposure to autophagy inhibitor. Prolonged exposure to an autophagy inhibitor halts autophagy at early cleavage stages, ultimately leading to developmental arrest. Two-cell mouse embryos were exposed to 75 µM of chloroquine (CQ) treatment for 14 and 48 hours. No CQ treatment was included in the control condition. Black arrowheads indicate preimplantation embryos that were arrested at the 2- and 4- cell stages. White arrowheads indicate preimplantation embryos that were developmentally regressed. Created with BioRender.com. (Image courtesy of Zuleika C. L. Leung).

Overall, a common practice of evaluating autophagic changes is via an autophagy flux assay with the use of an autophagy inhibitor that block autophagosome maturation or degradation. The inclusion of such autophagy inhibitor in experimental design ultimately results in the accumulation of autophagosomes in the cytoplasm. This enables the evaluation of changes in autophagosome dynamics in various experimental groups, providing a more accurate depiction of changes in autophagy levels. In preimplantation embryos, prolonged exposure to autophagy inhibitors halts autophagy at early preimplantation stages which ultimately leads to developmental arrest and cell death (Figure 3) as autophagy plays a vital role in preimplantation development. Thus, researchers must take caution when including autophagy inhibitors in their preimplantation embryo studies, ensuring the inhibitors are used at a sufficient level and time-period of exposure to properly evaluate autophagy but not induce cell death.

Past studies have employed various ways to quantitatively measure autophagy levels in preimplantation embryos. Immunofluorescence (IF) staining of autophagic markers like LC3, ATG5, and p62 are commonly utilized. In addition, methods like transcriptome analysis, Western blot, transgenic mice, and autophagy-monitoring probes are also applicable in evaluating autophagy. Here, we summarize various methods of assessing autophagy in mammalian preimplantation embryos.

### 4.1. 3.1 mRNA transcript analysis of autophagy markers

The central dogma of biology outlines that protein products are a result of mRNA translation in the cell. Thus, analyzing mRNA levels of autophagy markers reveal transcript profiles of preimplantation embryo autophagy. Quantitative polymerase chain reaction (qPCR) is a simple yet powerful method of analyzing mRNA transcript abundance. It has been employed in past literature to identify basal levels of mRNA transcripts of autophagy markers like *Atg5, Atg6*, and *Lc3* at each preimplantation stage [46,48]. qPCR is a feasible assay for investigating preimplantation embryo autophagy levels because qPCR only requires a small amount of mRNA, which preimplantation embryos can easily provide. Through polymerase chain reaction, qPCR generates additional copies of the target mRNA as cDNA to measure the expression levels of the target transcript. Another advantage is that reagents for qPCR are highly accessible. Primers of autophagic markers are commercially available, and customization can be easily achieved with the help of public databases like basic local alignment search tool (BLAST). Although qPCR can provide significant insights into mRNA transcript levels, it only reveals relative transcription levels and thus qPCR does not provide an exact quantification of mRNA expression of autophagy markers. Additionally, caution must be taken when choosing a reference gene for qPCR studies. Due to the dynamic changes in blastomere number, cell volume, and developmental progression at each cell stage, commonly used housekeeping genes like *β-actin* are not reliable genes for normalization. Previous studies identified that species-specific reference genes like *Gapdh* (glyceraldehyde 3-phosphate dehydrogenase) are stably expressed in all stages of preimplantation embryos [69–71]. Although these suggested reference genes are determined to be more reliable for normalization, the authors also suggested the use of multiple reference genes to increase the power of statistical analyses [69–71]. However, measuring multiple reference genes can quickly exhaust embryo samples and thus larger embryo pools are required for this method. Alternatively, the inclusion of an exogenous reference mRNA can also enable the normalization of target gene expression. It is important that the exogenous reference mRNA incorporated into embryo samples is not endogenously expressed in the embryos, and thus careful selection of exogenous gene products is required. Our group has previously employed luciferase mRNA products in embryo pools based on embryo numbers [46]. This method enables the normalization of target mRNA expression to embryo numbers; however, embryo samples must be of the same preimplantation stage to avoid stage-specific differences.

The increasingly popular technique of single-cell RNA sequencing can also be used to measure autophagy markers in preimplantation embryos. A newly published study by Song et al. [72] was the first to conduct a single-cell transcriptomic analysis of autophagy markers in human preimplantation embryos. The authors revealed that autophagy is constitutively active throughout preimplantation development, but autophagy-related gene expressions are tightly regulated epigenetically at each preimplantation stage [72]. Specifically, the *Atg* gene family is expressed at a much higher level beginning at the blastocyst stage, indicating the utilization of autophagy turnover for cell fate determination [72]. Transcriptomic analysis is an increasingly popular method of mRNA analysis as it reveals a great amount of information in mRNA transcript products on a single-cell basis. One advantage of this method is that it is not limited by the small number of blastomere or cells in a developing embryo. mRNA profiles of preimplantation embryos at the early stages like the zygotic and 2-cell stage can be easily assessed, in contrast to qPCR that requires a detectable level of mRNA abundance in the embryos. Although single-cell transcriptomics can provide a substantial amount of information, data analysis can be overwhelming and require an extensive process of manipulation. Adil et al. [73] extensively summarized the computational and technical challenges to single-cell transcriptomic analysis, highlighting the complexity of “big data” analysis. Another downside of single-cell transcriptomic analysis is that this method is relatively expensive. The cost can easily exponentiate especially when transcriptomic analyses of multiple samples are required to make inferences accurately.

However, autophagy evaluation in preimplantation embryos based solely on mRNA levels is insufficient. Comparing the mRNA abundance of autophagic markers only reveals basal levels and fluctuations in autophagy levels. Autophagic turnover cannot be determined due to the inability to measure autophagosome flux using mRNA transcripts. Although mRNA levels reveal important information about the transcriptional profile of autophagic markers in embryos, autophagy pathway constituents can only take part in autophagy as a protein complex. Some even require post-translational modifications and activation to become fully functional. Thus, analysis of protein expression and localization would be crucial in understanding autophagy in preimplantation embryos.

### 4.2. Immunofluorescence (IF) staining of autophagy markers

The use of immunofluorescence (IF) staining as means of presenting protein expression of autophagic markers has been extensively employed. IF uses immunofluorescence-linked antibodies that recognize target proteins to detect their presence and expressions. The protocol for IF staining of preimplantation embryos is relatively similar to other cell types. However, the staining procedure must be carefully executed as embryos are frail and are prone to mechanical wear and tear. For example, the use of glass coverslips on glass microscope slides is common practice in other cell types but cannot be applied to preimplantation embryo samples. The suspension of preimplantation embryos between glass slides and coverslips often compresses the embryos, and thus damages the structural integrity. Alternatives like glass-bottom dishes must be employed to preserve the structure of the preimplantation embryos.

Although IF staining has been carried out in a similar manner in most studies, the methods of analyzing IF results vary. The primary goal of IF staining is to identify the localization and levels of protein markers in the embryos. Various types of imaging modalities in microscopy are applicable to monitoring embryo dynamics [74]. These imaging techniques enable capturing of images that reveal the localization of proteins. These images can also be analyzed for fluorescence signals at specific focal points of the 3-dimensional embryo. It is important to note that preimplantation embryos are round and thus a series of images must be captured at multiple panes of focal points as a “z-stack” to capture the localization of autophagosomes more accurately across the entire embryo. The number of images in a z-stack is variable and up to researchers’ preferences. Of course, the more images captured, the more accurate the estimation would be. However, it is nearly impossible to image every autophagosome in an embryo due to their small sizes (0.5 to 2 µm; [75]). Additionally, photobleaching, the fading of fluorescent signal, can be a problem if the embryos are exposed to the light source of the microscope for a prolonged period [76,77]. Researchers must carefully account for the complication of losing fluorescence signals if a high number of images are to be captured.

In addition to the localization of autophagic markers, images can also be quantitatively analyzed for the level of autophagy-associated proteins in preimplantation embryos. Multiple different strategies were employed in previous studies. First, the measurement of LC3 puncta count. LC3 puncta are the most measured autophagic marker because they are highly representative of autophagosomes and are readily quantified. LC3-II, which originated from their precursor LC3 and LC3-I, localizes to autophagosomes as they are formed and are consumed along with the autophagosomes through lysosomal degradation. Thus, LC3-II can be counted to quantify the amount of autophagosomes present [78]. The method of counting is up to the researcher; however, manual counting is not recommended as it is prone to error and extremely labor exhaustive. Another important aspect of autophagosome counting in embryos is cell number. The level of autophagy varies across different cell stages and the number of LC3 puncta multiplies as embryos progress through developmental stages [46]. Simply measuring puncta count or fluorescence signal to measure autophagy levels is limited as drastic changes in blastomere number and cell volume across developmental stages occur in the preimplantation embryo. Thus, by normalizing to cell numbers, the level of autophagy can be presented on a “per cell” basis, providing a more accurate estimation of autophagy in preimplantation embryos. Similarly, manual counting of cell numbers is common but labor intensive.

**Figure 4.**
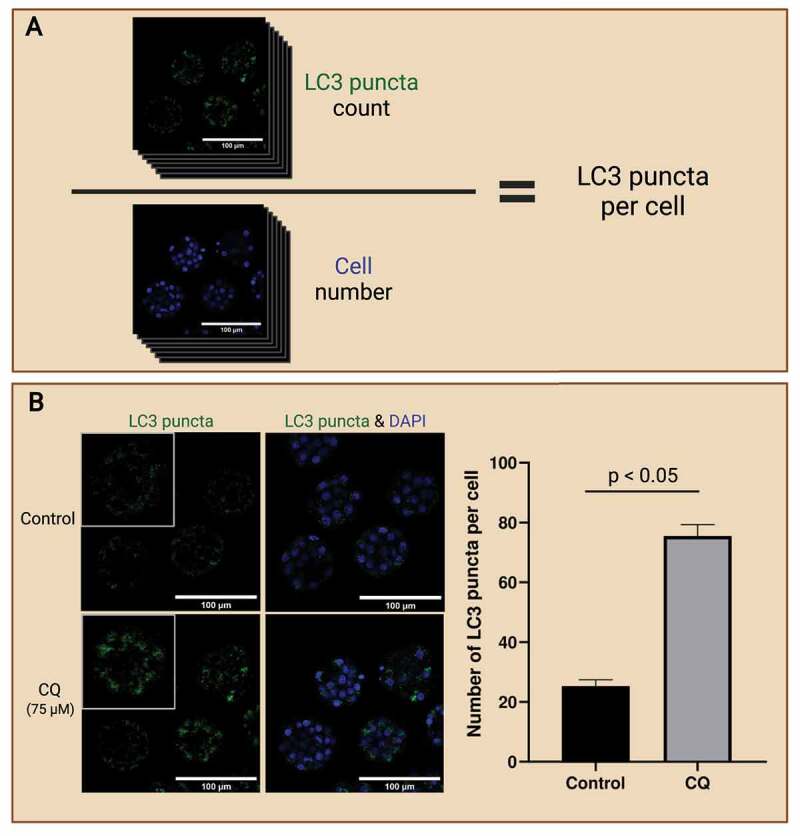
LC3 puncta count to access autophagy in preimplantation embryos. Workflow of analyzing LC3 puncta abundance to reflect autophagy levels in preimplantation embryos (A). Preimplantation mouse embryos were immunofluorescence-stained for LC3 puncta and counterstained with DAPI for cell nuclei. The whole embryo was captured on a confocal microscope; then LC3 puncta (top) was counted, and cell number (bottom) was identified. LC3 puncta count can be normalized to the cell number to present accumulation in a per-cell basis. Autophagic flux assay enables the evaluation of autophagic changes in preimplantation embryos with the use of an autophagy inhibitor (B). One example of an autophagy inhibitor is chloroquine (CQ), which inhibits autophagosome degradation by neutralizing hydrolytic enzymes in the lysosomes. Mouse preimplantation embryos were cultured for 40 hours, then treated with 75 µM of CQ for 30 mins prior to fixation. Note that treatment with CQ resulted in the accumulation of LC3 puncta (green), which reveals the buildup of autophagosomes in the preimplantation embryos after halting autophagosome degradation. An example bar graph shows the expected results of LC3 puncta accumulation per cell in inhibitor-treated vs. non-inhibited control embryos (p<0.05). No CQ treatment was included in the control condition. The difference in LC3 puncta count per cell between these groups reveals the changes in the rate of autophagosome formation and degradation. Scale bars = 100 µm. Created with BioRender.com. (Image courtesy of Zuleika C. L. Leung)

Alternatively, the use of computer algorithms in counting autophagosomes and cell numbers in preimplantation embryos may provide benefits over manual counting. When counting autophagosome numbers, selection criteria must be established prior to counting. For example, autophagosomes may be counted based on size. The quantification of fluorescence signal intensity is also commonly employed when measuring LC3-II expression. This is because previous studies have identified that LC3 puncta are likely clustered together when autophagy is inhibited by autophagy inhibitors like CQ [66]. In this case, many clusters of LC3 puncta may be disregarded as they were excluded from the selection criteria. Thus, many studies opt to measure fluorescence signal intensities to prevent such errors. This method is ideal for measuring autophagic flux in preimplantation embryos, but it is highly susceptible to background signals. On the other hand, computer algorithms that accurately detect cell counts in preimplantation embryos are becoming more readily available and user-friendly. One such example is the MINS program developed by Lou et al. [79]. The program is shown to be efficient in detecting preimplantation embryos at various developing stages, with at least 82% accuracy in determining cell numbers as well as identifying ICM and trophoblast cells [79]. The use of computer algorithms provides a vast advantage in efficiency as it greatly reduces the time spent on manual counting, especially of blastocyst stage embryos which consist of a large cell population and volume. It is vital that the whole embryo is captured with the same image capture settings, with minimal distances between each image of the z-stack so that the cell population can be accurately identified. Figure 4 depicts example images of preimplantation mouse embryos after immunofluorescence staining of LC3 puncta and counter-stained for cell nuclei.

In addition to LC3-II, other autophagic markers like p62 and ATG5 can also be assessed by IF staining. P62 is an adaptor protein that brings ubiquitinated proteins into autophagosomes for degradation. It is an important autophagy marker as it reflects the level of autophagy specifically related to ubiquitinated protein degradation. ATG5 is an initiator protein for autophagosome formation, thus its expression level is representative of the level of autophagosome formation. The utilization of autophagy inhibitors also enables the evaluation of autophagy flux by autophagic marker turnover through fluorescence intensity levels. Careful analysis of these markers must be conducted because p62 and ATG5 are present in the cytoplasm regardless of participation in the autophagy pathway, thus increase fluorescence intensity of these markers may not necessarily suggest an elevation in autophagy.

### 4.3. Western blot (WB) analysis of autophagy markers

Western blot (WB) is an essential assay for measuring levels of autophagy markers, but this assay is less often employed in preimplantation embryos due to the limited amount of protein present in the embryos. Regardless, WB favorably allows for the measurement of multiple autophagic markers with the same embryo sample. Additionally, the method of membrane stripping and re-probing enables the measurement of other markers of similar sizes using the same membrane; though antibody stripping may possibly remove protein samples on the blot [80].

**Figure 5.**
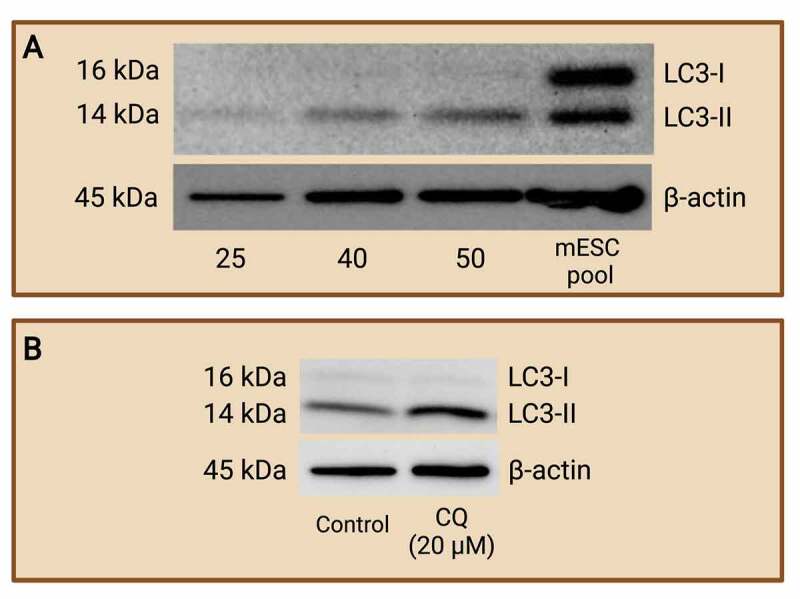
Western blot analysis of LC3-I and LC3-II protein expression to monitor autophagy. Mouse preimplantation embryos were cultured to the blastocyst stage and separated via electrophoresis with various numbers of blastocyst per lane. Western blot analyses of LC3-I and LC3-II were carried out to determine the minimum number of blastocysts required to achieve LC3 abundance at a detectable level. R1 wild-type mouse ES cells pool was included as a control. It was determined that a minimum of 50 blastocysts is required (A). An example of western blot of LC3-I and LC3-II after chloroquine (CQ) treatment (B). Mouse preimplantation embryos were cultured for 48 hours, then treated with 20 µM of CQ for 30 mins prior to fixation. Note that treatment with CQ resulted in a greater abundance of LC3-II (14 kDa) compared to control embryos, which reveals the buildup of autophagosomes in the preimplantation embryos after halting autophagosome degradation. The difference in LC3-II abundance between groups reflects autophagic flux that can be easily compared by densitometry analysis. No CQ treatment was included in the control condition. LC3-I (16 kDa); LC3-II (14 kDa); and β-actin (45 kDa; loading control) are shown. Created with BioRender.com. (Data courtesy of Zuleika C. L. Leung).

WB is seldomly conducted using preimplantation embryos. Past literature has employed this method to one, monitor autophagy levels; and two, to evaluate autophagic flux by quantifying autophagic protein turnover in the preimplantation embryos after exposure to an autophagy inhibitor that prevents the maturation or the degradation of autophagosomes. Song et al. [81] successfully measured apoptotic protein expression through WB, but the authors employed around 200 bovine embryos per lane. Similarly, Zamfirescu et al. [82] used 100 embryos per lane to quantify the protein expression of mTOR, an upstream autophagy regulator, in mouse preimplantation embryos. These studies demonstrate that a large embryo pool must be included in the WB assay and thus it quickly exhausts embryo samples. Our lab recently established that the measurement of the autophagy marker, LC3-II, requires at least 50 mouse blastocysts per lane (Figure 5A). Additionally, embryos treated with autophagic inhibitors like CQ can also be easily compared and analyzed for autophagic flux through densitometry analysis (Figure 5B). We propose that the use of WB assay in quantifying autophagy pathway protein constituents is probable and advantageous but may not be feasible due to the large number of preimplantation embryos required for this assay.

### 4.4. Autophagy probes and transgenic models

**Figure 6.**
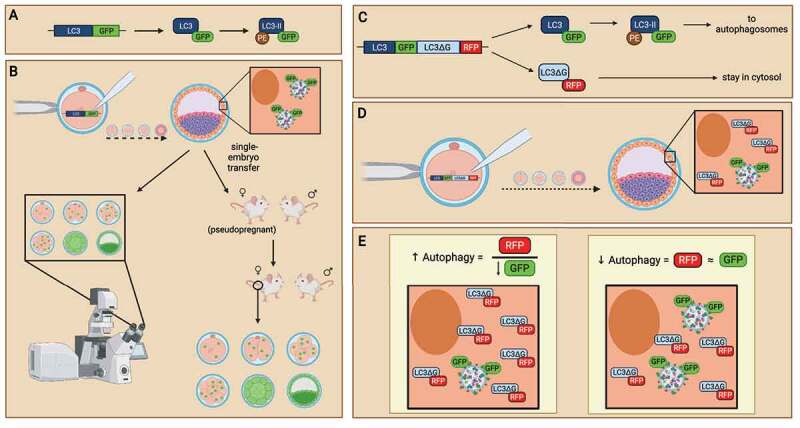
The use of LC3-monitoring probes in evaluating autophagy in preimplantation embryos. Probes of LC3 followed by green fluorescence protein (GFP) are generated (A) and introduced into preimplantation embryos as an internal reporter for autophagic activity (B). mRNA probe of GFP-LC3 is microinjected into embryos to monitor autophagy levels in preimplantation embryos. When preimplantation embryos activate autophagic mechanisms, GFP-tagged LC3 will be translated and incorporated into autophagosomes, allowing the detection of autophagy levels at various cell stages (left). DNA probe of GFP-LC3 is microinjected into embryos to create transgenic mice. Embryos expressing the GFP-LC3 probes can be transferred into pseudopregnant mice to create transgenic animal models which readily express GFP-LC3 (right). Similar to GFP-LC3 probes, the GFP-LC3-RFP-LC3∆G probe contains a GFP-tagged LC3, except that an RFP-LC3∆G sequence immediately follows to translate a red fluorescence protein (RFP)-tagged LC3 with modification to its C-terminal glycine subunit (C). The GFP-LC3-RFP-LC3∆G probe can be introduced to preimplantation embryos by microinjection at the zygotic stage. Both GRP-LC3 and RFP-LC3∆G are co-translated (D). GFP-LC3 is expressed with green fluorescence and participates in autophagy. In contrast, RFP-LC3∆G remains in the cytosol due to the modification to the LC3 protein. This GFP-LC3-RFP-LC3∆G probe enables the evaluation of each step of the autophagy pathway by measuring the GFP and RFP expression. Analysis of the ratio of RFP:GFP reflects the turnover of autophagosomes, in which the increase of RFP:GFP (by decreasing GFP) ratio is indicative of autophagy induction (E). Created with BioRender.com.

As mentioned earlier in the section, the use of autophagy inhibitors still has its limitations. These limitations points to the development of an alternate method of measuring autophagy – autophagy-monitoring probes. Usually, autophagy probes are mRNA probes generated in which autophagy genes are conjugated with fluorescent proteins. Using the internal cell machinery, these autophagy genes translate into proteins, along with fluorescent protein conjugates, prior to their participation in the autophagic pathway. Past literature has generated and employed these probes in the study of autophagy levels in the preimplantation embryos [83,84]. One of these probes simply consists of green fluorescent protein (GFP)-tagged LC3 (GFP-LC3) (Figure 6A). GFP-LC3 probes were injected into zygotes by microinjection and these embryos were incubated to various cell stages *in vitro*. The authors identified that these probes do not interfere with normal autophagic mechanisms nor impact blastocyst development [83]. The use of the GFP-LC3 probes enabled the researchers to visually evaluate autophagy at each cell stage, which provided significant information as the basal level of autophagy is relatively low. The use of these probes also enables the ability to conduct live-cell visualization of autophagy in the preimplantation embryos (Figure 6B). This method provides advantages in which autophagy turnover, by GFP-LC3 expression, can be evaluated throughout development.

Although GFP-LC3 probes enable researchers to evaluate the dynamics of autophagy in preimplantation embryos, it doesn’t provide an extensive evaluation of each step in the autophagic pathway. An improvement in the GFP-LC3 probe is the GFP-LC3-RFP-LC3∆G probe [84]. GFP-LC3-RFP-LC3∆G probes enable the visualization of autophagosome participation in the autophagic pathway. Essentially, a GFP is conjugated to LC3 mRNA while red fluorescence protein (RFP) is conjugated to a modified LC3 mRNA where the LC3 mRNA lacked a C-terminus glycine (Figure 6C). The GFP-LC3 mRNA was then fused with an RFP-LC3∆G sequence, creating the GFP-LC3-RFP-LC3∆G probes. The authors introduced these probes into mouse oocytes by microinjection, then through *in vitro* fertilization, mouse embryos expressing both GFP and RFP were formed [84]. When autophagy is lipidated, intrinsic ATG4 proteases cleave between GFP-LC3 and RFP-LC3∆G. GFP-LC3 is expressed with green fluorescence and participates in autophagosome formation after lipidation by PE. When autophagosomes are degraded by lysosomes, GFP-LC3 is also degraded, and the green fluorescence in the autophagosome is diminished. On the other hand, RFP-LC3∆G remains in the cytosol, unable to incorporate into autophagosomes due to the modification to the LC3 protein at the C-terminal (Figure 6D); red fluorescence thus remains in the cytosol. In brief, when autophagosome forms, cells would be expressed in yellow fluorescence due to the co-expression of GFP and RFP; but when autophagosomes are degraded, cells would express in red fluorescence only, as GFP is degraded through autophagy while only RFP persists. This GFP-LC3-RFP-LC3∆G probe enables the evaluation of each step of the autophagy pathway by measuring the GFP and RFP expression. Analysis of the ratio of GFP:RFP over various time points of development reflects the turnover of autophagosomes. It also enables the measurement of autophagic flux without the use of autophagy inhibitors (Figure 6E). These autophagy-monitoring probes possess many advantages in evaluating autophagy in preimplantation embryos. However, one disadvantage of these probes is that embryo microinjections are highly technical and require a great amount of precision. As embryos are delicate, personnel performing microinjections must be highly skilled to minimize the stress caused by embryo manipulation.

Another method of evaluating autophagy in preimplantation embryos is the generation and use of transgenic models [7,85]. The generation of transgenic mice was executed through the transfer of blastocysts, after microinjection with the autophagy-monitoring probes mentioned above, to pseudo-pregnant mice. The incorporation of these DNA transgene probes in the embryos enables the expression of GFP-tagged LC3. Embryos are grown to birth and pups are weaned and genotyped for the presence of GFP-LC3 transgene. After genotyping, female mice that express the GFP-LC3 transgene were mated with wild-type male mice, creating embryos that are heterozygous for the autophagy-monitoring probes. With the expression of autophagy-monitoring probes, these embryos can be used to evaluate autophagy at various cell stages of preimplantation development (Figure 6B). Limitations of this method include the lengthy and costly generation of mouse colonies. Overall, the use of autophagy-monitoring probes is a viable technique for evaluating preimplantation embryo autophagy. Its use is broadly applicable to a variety of autophagy experiments, for example, the evaluation of autophagy after embryo treatment exposure or genomic manipulations.

### 4.5. Embryonic stem cell and trophoblast stem cell models

While preimplantation embryos act as an excellent model for studying cellular mechanisms, researchers often find limitations in embryo studies as the number of gene products and proteins is limited, especially at the early preimplantation stages. It is no doubt that researchers are challenged by this nature when evaluating autophagy levels in the preimplantation embryo. As such, studies turn to the use of embryo-derived stem cell lines, especially embryonic stem cells (ES cells) and trophoblast stem cells (TS cells) as an alternate model for embryo experiments. In brief, ES cells are generated by isolating ICM from a blastocyst *in vitro* [86]. With the appropriate nutrients and supplementing culture conditions, ES cells can continuously proliferate while retaining their pluripotency. Similarly, TS cells are generated *in vitro* from isolated trophectoderm in a blastocyst [87]. These cells can remain undifferentiated while retaining the potential for differentiating into various trophoblast cell types. ES cells and TS cells act as excellent models for preimplantation embryos because of their “stemness”. Essentially, these cells are considered immortal and can easily proliferate given the appropriate culture conditions and supplementations. Their ability to regenerate poses advantage in scientific research because these stem cells can provide a tremendous amount of information when utilized in experimental studies. The number of cells that can be grown in a dish is exponential compared to the number of blastomeres in a blastocyst. Additionally, the use of embryo-derived stem cells of human origin enables the investigation of autophagy via methodologies that are impractical in human embryos. The conservation of the autophagic pathway in human embryos (or embryo-derived stem cells of human origin) may be comparable to the autophagic pathway of other mammalian models. Thus, the use of stem cells opens doors to many available assays and advanced techniques that cannot be easily employed in the preimplantation embryo.

**Figure 7.**
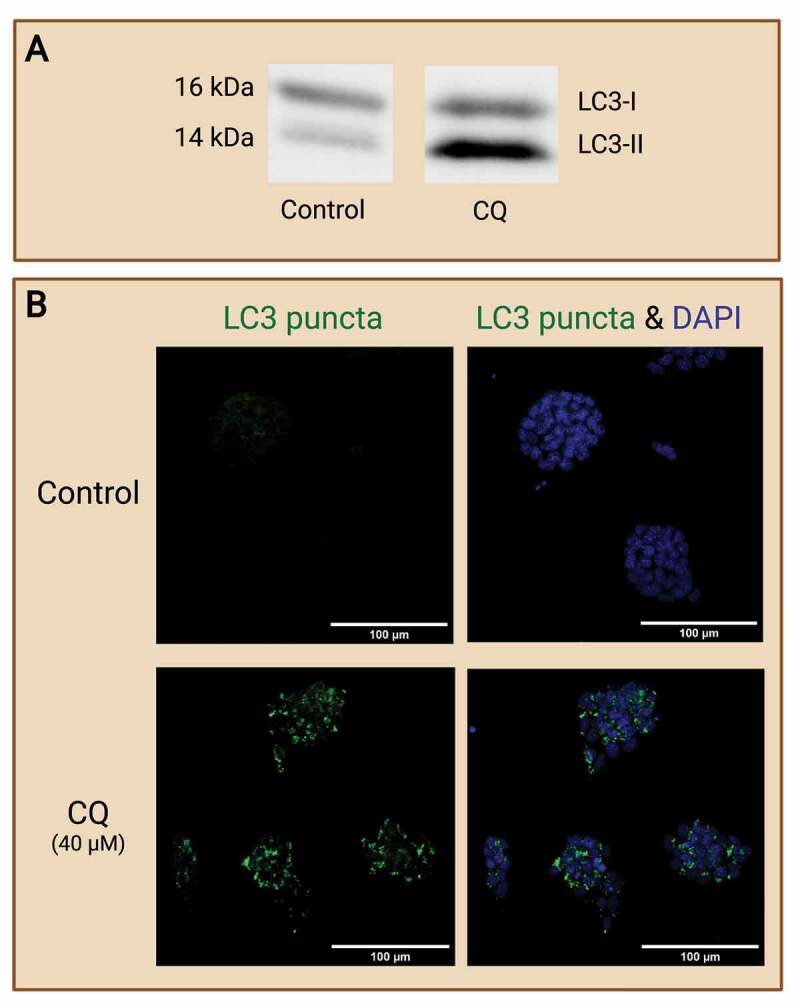
Evaluating autophagy in embryo-derived stem cell lines. Embryo-derived cell lines like embryonic stem (ES) cells and trophoblast stem (TS) cells are great models for evaluating autophagy in preimplantation embryos. ES cells and TS cells are derived from the ICM and trophoblast, respectively, from a developing blastocyst. These cell lines retain the typical expression of their original cell source and can thus provide potential avenues for focused investigation of embryonic cell type-specific effects. (A) Example of Western blot which shows changes of autophagy in wild-type mouse ES cells (R1 line; courtesy of Andras Nagy, Toronto), as reflected by the autophagic marker LC3-II (14 kDa), in response to chloroquine (CQ) treatment. No CQ treatment was included in the control condition. (B) Example images of IF staining of mouse ES cells to detect changes in LC3 puncta after CQ treatment. Note that treatment with CQ resulted in the accumulation of LC3 puncta (green), which reveals the buildup of autophagosomes in the ES cells after halting autophagosome degradation. No CQ treatment was included in the control condition. Scale bars = 100 µm. Created with BioRender.com. (Data and image courtesy of Hailey L. M. Hunter).

Certainly, the previously mentioned method of evaluating autophagy via mRNA transcript analysis, IF, WB, autophagy-monitoring probes, and transgenic models, are also applicable to studies of embryo-derived stem cell lines (Figures 7A and 7B). mRNA transcript analysis, IF, and WB are no longer limited by mRNA or protein abundance in the cells. However, cell lines developed *in vitro* likely experience epigenetic modifications in gene expressions due to adaptation to culture conditions [88], thus interpretation of results must be carefully conducted as potential cell-specific changes are possible. Past studies have demonstrated the ability to use flow cytometry to evaluate autophagosome formation in cells [89,90]. Markers of mitochondrial autophagy (also known as mitophagy) were also analyzed previously in various cell types [91]. It is no doubt that ES cells and TS cells can be employed via a similar methodology when analyzing the level of autophagosome formation and maturation. The use of flow cytometry in evaluating autophagy at each cell cycle is highly translatable to ES cells and TS cell lines as they model preimplantation embryos that are proliferating [89]. Furthermore, molecular techniques like CRISPR and the generation of transgenic ES cells and TS cells can be easily employed in cell line models in comparison to embryo models. Mizushima et al. [27] utilized the transfection method to generate *apg5* knockout (*atg5*^−/−^) ES cells and established the essential role of ATG5 in autophagosome formations in mammalian cell types. Tra et al. [92] utilized transduction of GFP-LC3 reporters in human ES cells to demonstrate the elevation of autophagy during differentiation and cell fate determination. These models reveal fundamental mechanisms of autophagy which are essential in preimplantation embryo development. The use of embryo-derived stem cell lines can also be easily manipulated to investigate autophagy-related gene knockout or knockdown impacts in preimplantation embryos.

## 5. Summary

Autophagy plays an important role in preimplantation embryo development as well as stress adaptation. It is imperative that the balance in autophagy must be maintained to support strong developmental competency and enable embryo survival. Modulations of the autophagy pathway have been studied in the past to minimize disruption in preimplantation embryo development. For example, acute induction of autophagy with rapamycin significantly increased blastocyst development, trophectoderm cell number, as well as the viability in *in vitro* produced bovine embryos [93]. Short-term induction of autophagy also relieved bovine embryos from ER stress, which may have contributed to the improvement in blastocyst viability observed in the study [93]. So far, no consensus is established regarding which direction of autophagy modulation is of most benefit for successful preimplantation embryo development and pregnancy. However, the ability of autophagy modulation to improve early embryo development suggests future directions in investigating preimplantation embryo autophagy. This review also discussed various methods in evaluating autophagy in the context of preimplantation embryos, from mRNA transcript analysis to the analysis of protein expression of autophagic markers. We additionally included the utilization of embryo-derived embryonic and trophoblast stem cell populations as alternate cell models to expand the library of techniques and methods for studying autophagy in preimplantation embryo. Researchers are encouraged to carefully select their route of investigation to evaluate autophagy in the preimplantation embryo settings. We are hopeful that future investigations in preimplantation embryo autophagy will expand our knowledge of embryo development that will lead to novel treatments for infertility.
